# Assessing clinical reasoning ability in fourth-year medical students via an integrative group history-taking with an individual reasoning activity

**DOI:** 10.1186/s12909-022-03649-4

**Published:** 2022-07-26

**Authors:** Jian-Han Lai, Kuan-Hao Cheng, Yih-Jer Wu, Ching-Chung Lin

**Affiliations:** 1 Department of Medicine, MacKay Medical College, New Taipei City, Taiwan; 2 Department of Medical Education, MacKay Memorial Hospital, No. 92, Sec. 2, Chung-Shan North Road, Taipei, Taiwan; 3 MacKay Medicine, Nursing and Management College, Taipei, Taiwan

**Keywords:** History taking, Clinical reasoning, Principal diagnosis, Key information, Medical record writing

## Abstract

**Background:**

The most important factor in evaluating a physician’s competence is strong clinical reasoning ability, leading to correct principal diagnoses. The process of clinical reasoning includes history taking, physical examinations, validating medical records, and determining a final diagnosis. In this study, we designed a teaching activity to evaluate the clinical reasoning competence of fourth-year medical students.

**Methods:**

We created five patient scenarios for our standardized patients, including hemoptysis, abdominal pain, fever, anemia, and chest pain. A group history-taking with individual reasoning principles was implemented to teach and evaluate students’ abilities to take histories, document key information, and arrive at the most likely diagnosis. Residents were trained to act as teachers, and a post-study questionnaire was employed to evaluate the students’ satisfaction with the training activity.

**Results:**

A total of 76 students, five teachers, and five standardized patients participated in this clinical reasoning training activity. The average history-taking score was 64%, the average key information number was 7, the average diagnosis number was 1.1, and the average correct diagnosis rate was 38%. Standardized patients presenting with abdominal pain (8.3%) and anemia (18.2%) had the lowest diagnosis rates. The scenario of anemia presented the most difficult challenge for students in history taking (3.5/5) and clinical reasoning (3.5/5). The abdominal pain scenario yielded even worse results (history taking: 2.9/5 and clinical reasoning 2.7/5). We found a correlation in the clinical reasoning process between the correct and incorrect most likely diagnosis groups (group history-taking score, *p* = 0.045; key information number, *p* = 0.009 and diagnosis number, *p* = 0.004). The post-study questionnaire results indicated significant satisfaction with the teaching program (4.7/5) and the quality of teacher feedback (4.9/5).

**Conclusions:**

We concluded that the clinical reasoning skills of fourth-year medical students benefited from this training course, and the lower correction of the most likely diagnosis rate found with abdominal pain, anemia, and fever might be due to a system-based teaching modules in fourth-year medical students; cross-system remedial reasoning auxiliary training is recommended for fourth-year medical students in the future.

## Introduction

Differential diagnosis is important for clinicians and involves a deeper higher order thinking process about the evaluation of history taking, physical examination, review of laboratory data, and diagnostic images that exceed memorization, facts, and concepts [1]. Koenemann et al. reported that clinical case discussions with peer-taught and physician-supervised collaborative learning formats could promote clinical reasoning in medical students [2]. However, we used problem-based learning (PBL) in schools to train the clinical reasoning process for fourth-year medical students using a module system base that could easily restrict students’ thinking in the same organ system.

Assessing a medical student’s ability concerning differential diagnosis is important; however, there is no consensus on the most effective approaches to evaluate these reasoning skills. Many efforts have been made to develop a valid and reliable measure of clinical reasoning ability, including key feature questions (KF), diagnostic thinking inventory (DTI) and script concordance (SC) tests. Charlin B et al. reported that the Script Concordance test is a simple and direct approach to testing organization and use of knowledge with machine-scorable [3]. Bordage G et al. developed an inventory of diagnostic thinking which measure two aspects of diagnostic thinking: the degree of flexibility in thinking and the degree of knowledge structure in memory [4]. Fischer MR et al. demonstrated a modified electronic key feature examination of clinical decision-making skills for undergraduate medical students [5].

Simulation-based testing methods have also been developed to meet the need for assessment procedures that are both authentic and well structured. Sutherland et al. allowed students to watch a video trigger and discuss their diagnostic reasoning with an examiner demonstrating that it could be assessed [6]. Fürstenberg et al. developed a clinical reasoning indicators history-taking scale to quantitatively assess clinical reasoning indicators during history taking in undergraduate medical students [7], which was deemed suitable for the assessment of fourth-year medical students’ clinical reasoning ability.

The objective structured clinical examination (OSCE) evaluation has proven to be a reliable and valid method for assessing the six competencies defined by the Accreditation Council for Graduate Medical Education (ACGME) in surgery. The competencies assessed by a well-constructed OSCE include patient care and medical knowledge as well as skills like data synthesis and the ability to list differential diagnoses [8]. This evaluation method was able to identify significant deficiencies in musculoskeletal examination skills and the diagnostic reasoning of fourth-year medical students based on principles of the hypothesis-driven physical examination [9]. Using a group OSCE format makes it possible to assess the individual ability of a large number of students without the usual time and expense needed [10]. As the fourth-year medical students in MacKay Medical College had no clinical reasoning curriculum except an integrated eight modules of divided organ systems PBL training and clinical diagnosis medicine and practice. In this study, we aimed to evaluate the M4 students’ clinical reasoning ability through group history taking with standardized patient (SP) in different clinical scenarios that mimic clinical conditions and then creating individual students’ most likely diagnosis with supporting data to assess their clinical diagnosis ability.

## Material and methods

### Problem identification and target needs of fourth-year medical students’ clinical reasoning ability before clinical practice

The medical curriculum of Taiwan medical schools including 2 years general and basic education of the department of medicine. The third-year curriculum is the integration of contents based on the general structure, functions, pathology and behaviors of a normal person. The fourth-year curriculum is the integration of contents based on the concise clinically relevant anatomical structure, functions, and behaviors of abnormal and diseased persons, as well as clinical knowledge and skills. The fifth- and sixth-year curriculum is the clinical medicine education. The curriculum at Mackay Medical College for fourth-year medical students is an integrated eight modules of divided organ systems including (1) introduction to clinical medicine and cardiovascular system, (2) pulmonary system and endocrine and metabolism, (3) gastrointestinal system, (4) brain and behaviors, (5) musculoskeletal system and integument system, (6) host defense and infection, (7) fluid, electrolytes, renal and genitourinary system, (8) hematology and oncology. In this M4 systemic module, PBL, clinical diagnosis medicine and practice is implanted and integrated into semesters for clinical reasoning training. This study conducted during the period from March 2019 to August 2019 in MacKay Medical College, the major question in this study is wanted to know the M4 students’ clinical reasoning ability through history taking with standardized patient in different clinical scenarios.

### Educational objectives and assessment method

Accurate diagnostic reasoning is the fundamental basis for ensuring patient care and safety; thus, the development of diagnostic reasoning is a key component of UGY medical education. We created a group history taking with individual reasoning activity at Mackay Medical College which is an elective pre-clerkship education course for fourth-year medical students, with the aim of training and assessing students’ clinical reasoning ability. To evaluate clinical reasoning ability, Haring et al. developed an observation tool that consists of an 11-item observation rating form and a post encounter rating tool which are both feasible, valid, and reliable to assess students’ clinical reasoning skills in clinical practice [11]. They also reported that by observing and assessing clinical reasoning during medical students’ history taking, general and specific phenomena could be used as indicators, including taking control, recognizing, and responding to relevant information, specifying symptoms, asking specific questions that point to pathophysiological thinking, placing questions in a logical order, checking agreement with patients, summarizing, and body language [12]. We modified the methods that include history taking and clinical reasoning each time we let four to five students visit a standardized patient with a clinical case to collect enough data, and then individually write down key information and make a correct differential diagnosis. For assessment of clinical reasoning, and to train their principal diagnosis ability, students were asked to list 15 items of key information for differential diagnoses and list the three most likely diagnoses, including the first. We didn’t use a standard test like modified electronic key feature examination (KF) or diagnostic thinking inventory (DTI) is due to Findyartini A. reported that higher DTI scores only reflect a greater familiarity with the thinking process in diagnostic reasoning and do not necessarily represent better diagnostic performance [13], and the modified electronic KF examination lack SP simulated clinical setting scenario with history-taking interaction.

### Study setting of the individual reasoning teaching program

Five R1 residents of internal medicine were recruited as teaching faculty in Mackay Memorial Hospital and requested to recognize the educational content and assess the trainees’ history taking performance and provide 30 minutes immediate feedback including the definition of this symptom, etiology, how to evaluate and collect key information, the initial diagnosis and treatment planning. We created five clinical scenarios: hemoptysis, abdominal pain, fever, anemia, and chest pain. The participants were divided into small groups consisting of four to five trainees per team. During the process of reasoning training, the trainees were expected taking turns to ask question while gathering clinical information from the standardized patients as well as acknowledge the symptoms and signs of the disease in each scenario. Documenting key words from history taking which requires as many as 15 clues, is also crucial to reflect students’ comprehension of a patient’s problems. From the patient’s information, the trainees were asked to differentiate the diagnosis with associated status and finally reach their most likely diagnosis along with two tentative diagnoses. After reasoning training, the residents who observed the trainees’ performance would be required to share their comments and experiences as well as resolve the trainee’s questions. The post-reasoning questionnaire, which was designed to focus on the satisfaction of the teaching program, provides an opportunity for the students to review and self-evaluate improvement in clinical reasoning and history taking through the reasoning program. The process and learning objectives of the reasoning training program for fourth-grade medical students are presented in Table 1.

**Table 1:** Group history taking with individual clinical reasoning activity for M4 students

Process	Subject Steps	Learning outcome focus
1. Include faculty	Training 5 residents as a teacher	Post-training teaching ability assessment
2. Select teaching materials	Create five clinical scenarios for group training	Scenarios including hemoptysis, abdominal pain, fever, anemia and chest pain
3 Group history taking with individual reasoning	Use this program to train M4 student’s history taking and clinical reasoning	Trainees write down 15 key information for clinical reasoning, one most likely with two tentative diagnosis including reasons
4. Group feedback	Post-training immediate feedback and clinical reasoning discussion	Residents shared their experience in the class and resolve trainee’s questions
5. Questionnaire	Post-training feedback questionnaire	Post-assessment: questionnaire for review improvement

### Participants and educational content

Fourth-grade medical students from MacKay Medical College were enrolled to participate electively in a system-based teaching program. We also trained five residents from MacKay Memorial Hospital as teachers to evaluate the trainees’ performances based on the checklists and then provide immediate feedback. Additionally, five standardized patients (SPs) who simulated the symptoms and signs of the teaching scenarios were also able to offer post-study remarks, all methods were carried out in accordance with relevant guidelines and the ‘Declaration of Helsinki’.

The reasoning teaching program was designed to provide an opportunity for fourth-grade medical students to perform history taking while gathering clinical information from the SPs as well as acknowledge the symptoms and signs of the disease in each scenario. Five patient scenarios were created as follows: lung cancer presenting with hemoptysis, acute pancreatitis presenting with abdominal pain, acute pyelonephritis presenting with fever, uterine myoma bleeding presenting with anemia, and acute coronary syndrome presenting with chest pain. The case settings of hemoptysis and chest pain were regarded as system-based thinking logics, whereas those with abdominal pain, fever, and anemia were multisystem-related. Each scenario highlighted different key words in the patient’s history, which could be clues for approaching the final and tentative diagnosis. The educational strategies for clinical reasoning in each scenario are presented in Table 2.

**Table 2:** Educational strategies of clinical reasoning in each scenario

Scenarios	Educational Strategies (1. Content; 2. Methods)
1. Hemoptysis	1. Understanding the symptoms including vital signs, the color of hemoptysis, timing, coagulopathy and associated conditions. 2. Practice to differ lung cancer (the most likely diagnosis), pulmonary TB and bronchiectasis; and then to recommend initial examination such as CXR, CBC, PT, PTT, Platelet and sputum smear, culture (AFB) and cytology.
2. Abdominal pain	1. Understanding the presentations including pain location, quality, provocation / palliative factors, region / radiation, timing and associated symptoms. 2. Practice to differ acute pancreatitis (the most likely diagnosis), acute cholecystitis and acute cholangitis; and then to recommend initial examination such as abdominal echo, white cell count, liver function test and amylase / lipase.
3. Fever	1. Understanding the symptoms including fever pattern, exclude upper airway, GI tract and GU tract infection and associated muscle, skin or autoimmune disease. 2. Practice to differ acute pyelonephritis (the most likely diagnosis), acute viral hepatitis and pneumonia; and then to recommend initial examination such as UA, white cell count, hepatitis work-up and CXR.
4. Anemia	1. Understanding the etiology of anemia including poor production, destruction and blood loss. 2. Practice to differ uterus myoma bleeding (the most likely diagnosis), GI tract bleeding and Vitamin B12 deficiency; and then to recommend initial examination such as CBC, MCV, serum iron and ferritin, arrange UGI endoscopy and colonoscopy and consult GYN evaluation.
5. Chest pain	1. Understanding the presentations including pain location, quality, provocation / palliative factors, region / radiation, timing and associated risk factors. 2. Practice to differ acute coronary syndrome (the most likely diagnosis), pleuritis and pneumothorax; and then to recommend initial examination such as12-lead EKG,cardiac enzyme, CXR and white cell count.

### Assessment and feedback

In this study, we focused on four major components: (1) the trainee’s group history taking score, (2) number of key information points, (3) diagnosis numbers and a correct most likely diagnosis rate, and (4) feedback questionnaire. The residents were capable of rating the scores of trainees using the checklist, based on observation of the overall performance of each trainee. By collecting the record paper at the end of this teaching program, we were able to accumulate the number of key information notes documented by each trainee. Meanwhile, from the record paper, we could check the number of diagnoses and whether they were correct or not. Moreover, a post-study questionnaire consisting of the degree of satisfaction with the reasoning teaching program, self-evaluation of ability and difficulty in clinical reasoning, and history taking was issued. The rubrics and questionnaire were based on a 5-point Likert scale for level of satisfaction.

### Statistical analysis

Data from the post-course feedback questionnaire and group history taking scores are shown as the mean ± standard deviation (SD). The correlations observed between the correct diagnosis group and the incorrect diagnosis group were analyzed using Student’s t-test. Statistical analyses were performed using SPSS 23.0 statistical package (SPSS, Chicago, IL, USA). All statistical analyses were based on two-sided hypothesis tests with a significance level of *p* < 0.05.

## Results

A total of 76 fourth-year medical students participated in this study, and five MacKay Memorial Hospital R1 residents were recruited as teachers in this clinical reasoning training program. Regarding the trainees’ score in group history taking with individual reasoning, the best score in the group history-taking scenario was chest pain (76.9%) and the worst was hemoptysis (50.1%), with an average group history-taking score of 63.5%. The number of key words the medical students were permitted to write down was between five and eight, and the average number was seven; fever had the highest number of key words in the scenarios (8), with the lowest number for abdominal pain (5). The average diagnosis number in each scenario was one to two; and the correct “the most likely diagnosis” rate ranged from 8.3 to 87.5%, with chest pain as the best scenario and abdominal pain as the worst (Table 3).

**Table 3:** Trainees’ scores in group history taking with individual reasoning activity

Scenarios (Tn)	Group History Taking Score	Key Information Number	Diagnosis Number	Correct “the most likely diagnosis” Rate
1. Hemoptysis (21)	50.1 ± 4.0	7.2 ± 1.5	1.7 ± 0.7	38.1%
2. Abdominal pain (12)	71.7 ± 2.5	4.6 ± 1.9	1.0 ± 0.9	8.3%
3. Fever (16)	66.2 ± 2.6	8.1 ± 0.7	0.6 ± 0.6	29.4%
4. Anemia (11)	56.7 ± 8.7	5.7 ± 0.6	1.0 ± 0.8	18.2%
5. Chest pain (16)	76.9 ± 4.8	7.7 ± 2.2	1.0 ± 0.4	87.5%
Average (76)	63.5 ± 11.4	6.9 ± 1.9	1.1 ± 0.8	38.2%

*Tn* Trainee’s number; Key Information Number, the written down number of 15 key words; Diagnosis Number, the accurate diagnosis number of three diagnosis answers

Concerning the post-history taking with individual reasoning feedback questionnaire, the overall satisfaction degree of the students with group history taking with reasoning was about 4.6/5 to 4.8/5. The teacher’s feedback and teaching ability were rated from approximately 4.7/5 to 4.9/5. The anemia score of the participants’ self-evaluation in these five scenarios was the most difficult in history taking (3.4/5) and clinical reasoning (3.5/5). Self-evaluation ability in history taking was worse for the abdominal pain (2.9/5) and anemia scenarios (2.8/5); the self-evaluation ability in clinical reasoning was also worst in the abdominal pain (2.7/5) and anemia scenarios (2.8/5) (Table 4).

**Table 4:** Post training feedback questionnaire of group history taking with individual reasoning

Scenarios	Hemoptysis	Abdominal pain	Fever	Anemia	Chest pain
Satisfaction degree with this workshop^a^	4.6 ± 0.5	4.6 ± 0.4	4.7 ± 0.5	4.8 ± 0.4	4.6 ± 0.5
Teacher’s feedback and teaching ability^b^	4.9 ± 0.3	4.9 ± 0.3	4.9 ± 0.2	4.7 ± 0.6	4.9 ± 0.3
Self-evaluation difficulty in history taking	3.1 ± 0.5	3.0 ± 0.4	3.2 ± 0.5	3.4 ± 0.5	3.1 ± 0.7
Self-evaluation difficulty in reasoning	3.0 ± 0.4	3.0 ± 0.7	2.9 ± 0.6	3.5 ± 0.7	2.9 ± 0.8
Self-evaluation ability in history taking^c^	3.0 ± 0.6	2.9 ± 0.7	3.0 ± 0.4	2.8 ± 0.8	3.6 ± 0.8
Self-evaluation ability in reasoning^c^	3.0 ± 0.5	2.7 ± 0.8	3.0 ± 0.7	2.8 ± 1.0	3.3 ± 0.8

The rubrics and questionnaire were based on the Likert scale for the Level of Satisfaction:

^a^very satisfied (5); satisfied (4); unsure (3); dissatisfied (2); very dissatisfied (1)

^b^Level of Agreement: strongly agree (5); agree (4); neither agree nor disagree (3); disagree (2); strongly disagree (1)

^c^Level of quality: excellent (5); very good (4); good (3); fair (2); poor (1)

Regarding ‘the most likely diagnosis’ after group history taking with individual reasoning, we divided the participants into ‘correct the most likely diagnosis group (*n*=29)’ and ‘incorrect the most likely diagnosis group (*n*=47)’ and found significant differences between these two groups, including the group history-taking score (*p* = 0.045), number of key words written down (*p* = 0.009), and diagnosis numbers (*p* = 0.004) (Table 5).

**Table 5:** The correlation between correct “the most likely diagnosis group” and incorrect “the most likely diagnosis group” in clinical reasoning (*n* = 76)

	Correct the most likely diagnosis group (*n* = 29)	Incorrect the most likely diagnosis group (*n* = 47)	*P* value
Group history taking score	66.8 ± 12.2	61.5 ± 10.5	0.045
Key information number	7.6 ± 1.9	6.4 ± 1.9	0.009
Diagnosis number	1.4 ± 0.6	0.9 ± 0.8	0.004

## Discussion

Clinical reasoning skills may help students to better focus on the efficient history taking and physical examinations that are required for making a correct diagnosis [14]. Williams DM et al. reported that a simulated clinic model which allowed one student to perform history taking and physical examination, and then following a small group collaboratively develop a prioritized differential diagnosis could support medical students’ basic and clinical science integration [15]. In our elective individual clinical reasoning training activity for M4 medical students, a group history taking with individual reasoning was based on educational strategies including five key scenarios integrated with R1 residents as teachers enabled to facilitate the clinical reasoning learning before clinical practice. This clinical reasoning teaching and assessment activity demonstrated that the M4 students needed remedial reasoning training, the teaching activity earned the students’ good satisfaction, and we also recorded these training courses as interactive e-learning education material for other medical students.

According to previous literature, Rencic et al. reported that most students enter clerkship with only poor (29%) to fair (55%) knowledge of key clinical reasoning concepts at US medical schools, thus developing clinical reasoning curricula to improve diagnostic accuracy and reduce diagnostic error is important [16]. Bonifacino et al. reported that exposure to a clinical reasoning curriculum was associated with both superior reasoning knowledge and written demonstration of clinical reasoning skills by third-year medical students on an internal medicine clerkship [17]. Harendza S et al. said that a longitudinal implementation of clinical reasoning in the curriculum would appear to be worthwhile on students’ case presentation and differential diagnostic skills [18]. For our M4 clinical reasoning curriculum in the future, we may construct self e-learning education materials including cross-system symptoms and signs.

Does the group history taking score reflect the clinical reasoning ability of medical students? Park et al. demonstrated that the clinical reasoning score was significantly correlated with diagnostic accuracy and grade point average but not with group history taking score or clinical knowledge score, and that some students might attain a high history taking score simply by asking and checking memorized items without adequate reasoning [19]. In our study, we found that the ability to correct “the most likely diagnosis” is very important, due to its correlation with history taking, identifying key information, and reasoning ability (Table 5). The purpose of group history taking is to increase more data of key information from the training group which can improve individual clinical reasoning, however, reasoning also depends on the background of individual knowledge and critical thinking ability.

Clinical reasoning is currently receiving much attention as its theory is complex and under-taught in both undergraduate and postgraduate education. The complexities of diagnostic reasoning and therapeutic concepts, including hypothesis generation, pattern recognition, context formulation, diagnostic test interpretation, differential diagnosis, and diagnostic verification, provide both the language and the methods of clinical problem-solving [20]. A challenge of teaching clinical reasoning at an undergraduate level is the lack of formal educational sessions [16]. The teaching should not be delayed until students fully understand anatomy and pathophysiology. Weinstein et al. successfully implemented a faculty development workshop for diagnosing and remediating clinical reasoning difficulties to help clinical teachers improve their skills [21]. In our study, we created a teaching faculty including R1 residents, and the R1 teacher gave students 30 minutes immediate group feedback post training which earned high satisfaction. This study has several limitations. First, it was performed in a single institution with only 76 fourth-grade students. Therefore, these results may not be generalizable to other institutions, which may have different clinical clerkship programs and student evaluation systems. Second, the authors tested only five case scenarios, which may not be sufficient to generalize these conclusions across a wider population. Third, the group history taking score used for the present analysis was based only on the group of patient encounters, not personal encounters.

In conclusion, formal teaching of the principles of diagnosis and the causes of diagnostic uncertainty and error is a fundamental requirement in medical education for the safety of doctors and patients. This can be delivered as an educational strand through the course of the program, starting with the principles of diagnosis, diagnostic uncertainty, and misdiagnosis in the first year. It can conclude with symptom-based and patient safety-focused diagnostic reasoning, along with strategies and interventions to manage diagnostic uncertainty safely and reduce the risk of diagnostic errors, in the final year. In this study, we developed auxiliary teaching material to improve diagnostic accuracy, including R1-residents as clinical reasoning teaching faculty and using group history with individual reasoning training. We created e-learning scenario videos to improve other students’ cross-system reasoning ability, which demonstrated high satisfaction.

## Data Availability

The datasets generated and/or analyzed during the current study are not publicly available due to this is a pilot study in M4 clinical diagnosis medicine practice for reasoning but are available from the corresponding author on reasonable request.

## Abbreviations

PBL: Problem-based learning
OSCE: Objective structured clinical examination
ACGME: Accreditation Council for Graduate Medical Education
SP: Standardized patient
